# Association of *NUCB2* genetic variants with the clinicopathological features of oral cancer

**DOI:** 10.7150/ijms.129479

**Published:** 2026-02-26

**Authors:** Chang-Chiang Yu, Hsueh-Ju Lu, Ming-Yu Lien, Chiao-Wen Lin, Jian-Hong Yu, Shun-Fa Yang, Chih-Hsin Tang

**Affiliations:** 1 School of Dentistry, China Medical University, Taichung, Taiwan.; 2 School of Medicine, Chung Shan Medical University, Taichung, Taiwan.; 3 Division of Hematology and Oncology, Department of Internal Medicine, Chung Shan Medical University Hospital, Taichung, Taiwan.; 4 School of Medicine, China Medical University, Taichung, Taiwan.; 5 Division of Hematology and Oncology, Department of Internal Medicine, China Medical University Hospital, Taichung, Taiwan.; 6 Institute of Oral Sciences, Chung Shan Medical University, Taichung, Taiwan.; 7 Department of Dentistry, Chung Shan Medical University Hospital, Taichung, Taiwan.; 8 Department of Orthodontics, China Medical University Hospital, Taichung, Taiwan.; 9 Institute of Medicine, Chung Shan Medical University, Taichung, Taiwan.; 10 Department of Medical Research, Chung Shan Medical University Hospital, Taichung, Taiwan.; 11 Department of Pharmacology, School of Medicine, China Medical University, Taichung, Taiwan.; 12 Department of Medical Laboratory Science and Biotechnology, Asia University, Taichung, Taiwan.; 13 Chinese Medicine Research Center, China Medical University, Taichung, Taiwan.

**Keywords:** nesfatin-1, *NUCB2*, oral cancer, genetic polymorphisms

## Abstract

Oral cancer ranks as the fourth most common cancer among men in Taiwan and the ninth most common cancer among men worldwide. Nesfatin-1, an adipokine derived from the precursor *NUCB2* gene, was originally discovered in hypothalamic neurons. The connections among lifestyle factors that promote cancer, *NUCB2* polymorphisms, and oral cancer are still not well understood. We examined the association of four *NUCB2* gene polymorphisms (rs1330, rs214101, rs757081, and rs10766383) and clinicopathological characteristics with oral cancer in Taiwanese men compared with healthy controls. According to our data, in patients aged ≥60 years, specific *NUCB2* genotypes were significantly associated with more aggressive disease features. Compared with the wild-type C/C genotype, carriage of at least one polymorphic allele (T allele at rs1330 or G allele at rs757081) was correlated with an elevated risk of progression to stage III/IV disease. Furthermore, the GA/AA genotypes at rs214101 and the TG/GG genotypes at rs10766383 were associated with elevated risks of both advanced-stage (III/IV) disease and lymph node metastasis. Our findings suggest that *NUCB2* SNPs may play a pivotal role in oral cancer progression and metastatic potential, particularly in older patients.

## Introduction

One of the key etiological factors of high death rates is oral cancer, which is the most frequent type of head and neck cancer 1. Oral cancer has emerged as one of the most prevalent malignant tumors worldwide, according to the "Global Cancer Statistics 2022" survey 1. Of all oral cancers, oral squamous cell carcinoma (OSCC) is the biggest general (representing nearly 90% of all malignancies of the mouth cavity) 2. Human papillomavirus infection and regular use of carcinogens including alcohol, tobacco, and betel nuts are major risk factors for OSCC 3, 4. Around 86% of Taiwanese oral cancer patients are frequent betel nut chewers 5, 6. Oral cancer is also linked with genetic anomalies caused by DNA repair, dysregulation of carcinogen metabolism and cell cycle regulation 7.

Adipokines are bioactive agents secreted by adipose tissue that regulate a range of physiological processes, including inflammation, homeostasis, insulin responsiveness, and immune responses 8. Metabolic and inflammatory pathways are influenced by key adipokines for instance nesfatin-1, adiponectin, resistin and leptin, which act locally or systemically through endocrine, autocrine, or paracrine processes 9. Adipokines have multifaceted effects in cancer, impacting the tumor microenvironment, cellular proliferation, apoptosis, angiogenesis, and metastasis 10-12. Originating from its precursor *NUCB2*, nesfatin-1 is an 82-amino acid polypeptide that was originally found in hypothalamic neurons 13. Additionally, peripheral tissues include adipose tissue, the pancreas, the ovaries, the colon, and the duodenum express nesfatin-1 14. Multifaceted roles have been related with nesfatin-1 and it is documented as an anorexigenic peptide having antihyperglycemic, anti-inflammatory and antioxidant functions 15, 16. Recently, investigations have found elevated *NUCB2* expression in colon, breast, endometrial, thyroid, and prostate cancers 17. These data reveal a clear link between nesfatin-1 level and poor prognosis, with the development of metastasis and reduced disease-free survival.

A change in a single nucleotide that takes place at a particular position in the genome is known as a single nucleotide polymorphism (SNP) 18. SNP distribution frequency comparisons between patient populations are commonly applied to estimate the prognosis and risk of diseases, including cancer 19, 20. No data are available on the links between carcinogenic lifestyle factors, *NUCB2* gene polymorphisms, and oral cancer. Consequently, this research investigated the impact of carcinogenic lifestyle factors and *NUCB2* gene polymorphisms on the likelihood of oral cancer development in a cohort of Taiwanese. We also investigated the relationships between *NUCB2* genotypes and the histopathological prognostic variables of oral cancer.

## Materials and Methods

### Study participants

We registered 1161 patients with oral cancer at Chung Shan Medical University Hospital in Taiwan. From the Taiwan Biobank Project, 1186 healthy controls (HCs) with no cancer history were randomly chosen and anonymized. Demographic data and carcinogenic lifestyle practices (alcohol use, cigarette smoking, betel nut chewing) were recorded. Daily smokers were defined as those who had smoked at least one cigarette per day for the three months prior. Alcohol consumers were defined as those who, on average, drank more than two alcoholic beverages per day. Oral cancer was assessed using the 2018 American Joint Committee on Cancer (AJCC) Cancer Staging Manual 21. A pathologist used the AJCC classification standards to evaluate tumor cell differentiation. Every method employed in the research involving human beings complied with the Declaration of Helsinki's standards. After gaining access to the data, each author reviewed and approved the study. The Chung Shan Medical University Hospital's Institutional Review Board approved the study before to its start.

### Selection and genotyping of SNPs

The *NUCB2* SNPs rs1330, rs214101, rs757081 and rs10766383 were selected based on previous reports 22, 23. Every SNP had a minor allele frequency greater than 5%. Genomic DNA was extracted from 3 mL peripheral blood samples using QIAamp DNA Blood Kits (Qiagen, CA, USA). Using previously described assessment techniques 8, 20, 21, allelic discrimination was performed on the SNPs. RT-qPCR experiments and the isolation of RNA were performed following the protocols we published earlier 24, 25.

### Statistical analysis

To assess the differences between the oral cancer and control groups, the Fisher's exact test and Mann-Whitney U test were employed, with *p*-values below 0.05 considered statistically significant. Logistic regression was used to calculate odds ratios (ORs) and their 95% confidence intervals (CIs) for the associations between genotype frequencies and oral cancer risk. The collected data were analyzed using version 9.1 of the Statistical Analytic System (SAS) software.

## Results

Table 1 shows the distribution of demographic and clinical characteristics in 1161 cancer-free HCs and 1,186 male patients with oral cancer. The percentage of patients aged ≥ 60 years was markedly higher in the oral cancer group (40.0%, n=464) than in the control group (34.9%, n=414) (*p* = 0.011). Controls reported betel quid chewing (*p* < 0.001), cigarette smoking (*p* < 0.001), and alcohol consumption (*p* < 0.001) markedly less frequently than the oral cancer patients (Table 1). The proportions of patients classified as T1/T2 or T3/T4 tumor status were similar. Regarding lymph node status, 66.3% of patients had N0 status, while 33.7% had N1-N3 status. The vast majority (99.5%) of patients were without distant metastasis (M0). Furthermore, 84.5% of patients had moderately or poorly differentiated oral cancer, compared to 15.5% with well-differentiated disease (Table 1).

Genotyping results for the *NUCB2* SNPs in HCs and oral cancer patients are presented in Table 2. The homozygous C/C genotype for rs1330 and rs757081, the homozygous G/G genotype for rs214101, and the homozygous T/T genotype for rs10766383, were the most common (Table 2). After adjusting for betel quid chewing, cigarette smoking, and alcohol consumption, none of the genotypes for the four *NUCB2* SNPs across different comparison groups exhibited a significant association with oral cancer (Table 2).

Next, we conducted a comparison of the distributions of clinical aspects and *NUCB2* genotypes among oral cancer patients. No significant influence of SNPs rs1330, rs214101, rs757081 and rs10766383 on clinicopathologic traits in oral cancer patients (Table 3-6). However, in patients aged ≥60 years, those carrying at least one polymorphic T allele at rs1330 (C/T + T/T genotypes) showed increased susceptibility to progression to stage III/IV disease compared with the C/C genotype (OR = 1.462; 95% CI: 1.011-2.114; *p* < 0.05) (Table 3). Similarly, the GA or AA genotypes at rs214101 were linked with a higher risk of stage III/IV disease (OR = 1.637; 95% CI: 1.102-2.432; *p* < 0.05) and lymph node metastasis (OR = 1.616; 95% CI: 1.067-2.446; *p* < 0.05) in patients aged ≥60 years with oral cancer (Table 4). In addition, individuals with at least one polymorphic G allele at rs757081 (C/G + G/G genotypes) exhibited greater susceptibility to stage III/IV disease compared with the C/C genotype (OR = 1.548; 95% CI: 1.070-2.239; *p* < 0.05) (Table 5). Furthermore, the TG or GG genotypes at rs10766383 were linked with an elevated risk of stage III/IV disease (OR = 1.748; 95% CI: 1.160-2.632; *p* < 0.05) and lymph node metastasis (OR = 1.963; 95% CI: 1.207-3.194; *p* < 0.05) in oral cancer patients aged ≥60 years (Table 6).

## Discussion

Many cancer reports have confirmed the effectiveness of biomarkers based on genetic aberrations related to tumors in assessing risk, aiding in early diagnosis, and predicting treatment results 26, 27. Approximately 1% of the overall population carries genetic polymorphisms, which are variations in genomic sequences among individuals. Repetitive sequences most frequently exhibit alterations in the form of SNPs 28. An expanding body of investigation has recently underscored the significance of SNPs and other genetic changes in defining and predicting pharmacotherapeutic functions in oral cancer 19, 29, 30. Moreover, the methodical identification of functional variants linked to cancer risk has demonstrated how SNPs in functional domains affect gene level and tumor susceptibility, highlighting the importance of SNPs in tumor biology 31. These findings are supplemented by thorough reviews that detail the biological and molecular processes through which SNPs affect gene expression, thereby affecting the progression and development of tumor 32. Hypothesis-driven genetic research has informed both case-control and prospective cohort reports that investigated the connection between SNPs and oral cancer. The studies have underscored the connection between changes impacting multiple biological mechanisms—for instance oxidative stress, DNA repair, and inflammation processes—and the evolution of oral cancer in patients 19, 33. We investigated polymorphisms in the *NUCB2* gene and noted their different distributions among oral cancer patients. Our investigation revealed that, in oral cancer patients aged ≥65 years, all four *NUCB2* SNPs (rs1330, rs214101, rs757081, and rs10766383) were significantly associated with advanced-stage (III/IV) disease. Furthermore, rs214101 and rs10766383 were also associated with lymph node metastasis.

Adipokines are unique bioactive peptides released by adipose tissues and play a role in various bodily functions 34. To examine the function of adipose tissue in the progression of inflammation and carcinogenesis, many investigators have been investigating this issue for the last two decades 35. Recent studies have documented that nesfatin-1 plays a pivotal effect in cell differentiation and the promotion of cancer cell death 17. High nesfatin-1 level was detected in colon, prostate, breast, and thyroid cancer tissues compared with surrounding non-cancerous tissues 36-38. Additionally, increased nesfatin-1 expression was strongly linked to metastasis and advanced tumor stage in renal cell carcinoma 39. A similar pattern of overexpression was also identified in colon and prostate cancer tissues 36, 40. By contrast, research on the relationship between *NUCB2* and oral cancer remains limited. We conducted this study to compare the allelic and genotypic distributions of *NUCB2* gene polymorphisms between HCs and patients with oral cancer. No significant difference was identified between the four *NUCB2* SNPs and overall oral cancer susceptibility. However, when we stratified oral cancer patients according to clinical parameters, all four *NUCB2* SNPs were significantly associated with advanced-stage (stage III/IV) disease in patients aged ≥65 years.

One of the main causes of cancer-linked mortality is metastasis. It is believed that lymphangiogenesis and the modification of preexisting lymphatics are crucial stages in metastasis 41. Higher numbers of lymphatic vessels are strongly associated with metastasis and clinical prognosis in a number of malignancies, and tumors can actively stimulate lymphatic expansion and lymphangiogenesis 25, 42. A poor prognosis and an increased probability of recurrence or metastasis are indicated by oral cancer with lymph node metastases 43, 44. Interestingly, in patients aged ≥60 years, specific *NUCB2* genotypes were markedly linked with lymph node metastasis. An age threshold of ≥60 years is frequently used in oral cancer research to identify elderly or geriatric patients in clinical and prognostic investigations 45. Despite having similar tumor biology, patients over 60 frequently show worse overall survival because to increased comorbidities, worse treatment tolerance, higher non-cancer-related mortality, and, occasionally, undertreatment 45. The GA or AA genotypes at rs214101 and the TG or GG genotypes at rs10766383 were linked with an increased risk of lymph node metastasis. It is important to note the limitations of the current study. More research is required, which calls for a larger sample size and a longer follow-up time. Furthermore, an independent cohort of oral cancer cases from Taiwanese communities and other cohorts found in open-access databases must be used to confirm the current findings.

To sum up, our study is the first to uncover links between *NUCB2* gene variants and oral cancer. In patients aged ≥60 years, specific *NUCB2* genotypes were significantly associated with more aggressive disease features. Compared with the wild-type C/C genotype, carriage of at least one polymorphic allele (T allele at rs1330 or G allele at rs757081) was associated with increased risk of progression to stage III/IV disease. Furthermore, the GA/AA genotypes at rs214101 and the TG/GG genotypes at rs10766383 were associated with elevated risks of both advanced-stage (III/IV) disease and lymph node metastasis. Our findings suggest that *NUCB2* SNPs may play a pivotal role in oral cancer progression and metastatic potential, particularly in older patients.

## Figures and Tables

**Table 1:** The distributions of demographical characteristics in 1186 controls and 1161 male patients with oral cancer.

Variable	Controls (N=1186)	Patients (N=1161)	p value
Age (yrs)			
< 60	772 (65.1%)	697 (60.0%)	p = 0.011*
≥ 60	414 (34.9%)	464 (40.0%)	
Betel quid chewing			
No	989 (83.4%)	385 (33.2%)	
Yes	197 (16.6%)	776 (66.8%)	p < 0.001*
Cigarette smoking			
No	554 (46.7%)	249 (21.4%)	
Yes	632 (53.3%)	912 (78.6%)	p < 0.001*
Alcohol drinking			
No	951 (80.2%)	720 (62.0%)	
Yes	235 (19.8%)	441 (38.0%)	p < 0.001*
Stage			
I+II		501 (43.2%)	
III+IV		660 (56.8%)	
Tumor T status			
T1+T2		569 (49.0%)	
T3+T4		592 (51.0%)	
Lymph node status			
N0		770 (66.3%)	
N1+N2+N3		391 (33.7%)	
Metastasis			
M0		1155 (99.5%)	
M1		6 (0.5%)	
Cell differentiation			
Well differentiated		180 (15.5%)	
Moderately or poorly differentiated		981 (84.5%)	

* p value < 0.05 as statistically significant.

**Table 2:** Odds ratio (OR) and 95% confidence interval (CI) of oral cancer associated with *NUCB2/nesfatin-1* genotypic frequencies.

Variable	Controls (N=1186) (%)	Patients (N=1161) (%)	AOR (95% C.I.)	p value
**rs1330**				
CC	466 (39.3%)	475 (40.9%)	1.000 (reference)	
CT	562 (47.4%)	515 (44.4%)	0.888 (0.724~1.090)	p=0.256
TT	158 (13.3%)	171 (14.7%)	1.141 (0.851~1.528)	p=0.378
CT + TT	720 (60.7%)	686 (59.1%)	0.942 (0.777~1.142)	p=0.544
**rs214101**				
GG	814 (68.6%)	777 (66.9%)	1.000 (reference)	
GA	322 (27.2%)	341 (29.4%)	1.038 (0.840~1.283)	p=0.732
AA	50 (4.2%)	43 (3.7%)	1.078 (0.664~1.751)	p=0.761
GA + AA	372 (31.4%)	384 (33.1%)	1.043 (0.852~1.276)	p=0.686
**rs757081**				
CC	475 (40.1%)	479 (41.3%)	1.000 (reference)	
CG	565 (47.6%)	510 (43.9%)	0.854 (0.696~1.048)	p=0.131
GG	146 (12.3%)	172 (14.8%)	1.262 (0.939~1.698)	p=0.123
CG + GG	711 (59.9%)	682 (58.7%)	0.935 (0.771~1.133)	p=0.491
**rs10766383**				
TT	317 (26.7%)	286 (24.6%)	1.000 (reference)	
TG	597 (50.3%)	586 (50.5%)	1.031 (0.819~1.297)	p=0.796
GG	272 (23.0%)	289 (24.9%)	1.173 (0.897~1.534)	p=0.245
TG + GG	869 (73.3%)	875 (75.4%)	1.075 (0.865~1.335)	p=0.514

The adjusted odds ratio (AOR) with their 95% confidence intervals were estimated by multiple logistic regression models after controlling for betel quid chewing, cigarette smoking, and alcohol drinking.

**Table 3:** Clinical statuses and genotypic frequencies of *NUCB2/nesfatin-1* rs1330 in 1161 oral cancer patients.

				NUCB2/nesfatin-1 rs1330					
	All (N=1161)			Age < 60 (N=697)			Age ≥ 60 (N=464)		
Variable	CC (N=475)	CT + TT (N=686)	p value	CC (N=268)	CT + TT (N=429)	p value	CC (N=207)	CT + TT (N=257)	p value
**Clinical Stage**									
Stage I+II	212 (44.6%)	289 (42.1%)	0.397	108 (40.3%)	184 (42.9%)	0.500	104 (50.2%)	105 (40.9%)	0.043^*,a^
Stage III+IV	263 (55.4%)	397 (57.9%)		160 (59.7%)	245 (57.1%)		103 (49.8%)	152 (59.1%)	
**Tumor size**									
≦ T2	240 (50.5%)	329 (48.0%)	0.390	128 (47.8%)	207 (48.3%)	0.900	112 (54.1%)	122 (47.5%)	0.155
> T2	235 (49.5%)	357 (52.0%)		140 (52.2%)	222 (51.7%)		95 (45.9%)	135 (52.5%)	
**Lymph node metastasis**									
No	315 (66.3%)	455 (66.3%)	0.997	163 (60.8%)	280 (65.3%)	0.235	152 (73.4%)	175 (68.1%)	0.210
Yes	160 (33.7%)	231 (33.7%)		105 (39.2%)	149 (34.7%)		55 (26.6%)	82 (31.9%)	
**Cell differentiation**									
Well	72 (15.2%)	108 (15.7%)	0.786	39 (14.6%)	65 (15.2%)	0.829	33 (15.9%)	43 (16.7%)	0.819
Moderate or poor	403 (84.8%)	578 (84.3%)		229 (85.4%)	364 (84.8%)		174 (84.1%)	214 (83.3%)	

* p value < 0.05 as statistically significant. ^a^ OR (95% C.I.): 1.462 (1.011-2.114) The odds ratio (OR) with their 95% confidence intervals were estimated by logistic regression models.

**Table 4:** Clinical statuses and genotypic frequencies of *NUCB2/nesfatin-1* rs214101 in 1161 oral cancer patients.

				NUCB2/nesfatin-1 rs214101					
	All (N=1161)			Age < 60 (N=697)			Age ≥ 60 (N=464)		
Variable	GG (N=777)	GA + AA (N=384)	p value	GG (N=467)	GA + AA (N=230)	p value	GG (N=310)	GA + AA (N=154)	p value
**Clinical Stage**									
Stage I+II	348 (44.8%)	153 (39.8%)	0.110	196 (42.0%)	96 (41.7%)	0.954	152 (49.0%)	57 (37.0%)	0.014^*,a^
Stage III+IV	429 (55.2%)	231 (60.2%)		271 (58.0%)	134 (58.3%)		158 (51.0%)	97 (63.0%)	
**Tumor size**									
≦ T2	391 (50.3%)	178 (46.4%)	0.203	225 (48.2%)	110 (47.8%)	0.930	166 (53.5%)	68 (44.2%)	0.057
> T2	386 (49.7%)	206 (53.6%)		242 (51.8%)	120 (52.2%)		144 (46.5%)	86 (55.8%)	
**Lymph node metastasis**									
No	523 (67.3%)	247 (64.3%)	0.311	294 (63.0%)	149 (64.8%)	0.637	229 (73.9%)	98 (63.6%)	0.023^*,b^
Yes	254 (32.7%)	137 (35.7%)		173 (37.0%)	81 (35.2%)		81 (26.1%)	56 (36.4%)	
**Cell differentiation**									
Well	124 (16.0%)	56 (14.6%)	0.542	71 (15.2%)	33 (14.3%)	0.766	53 (17.1%)	23 (14.9%)	0.554
Moderate or poor	653 (84.0%)	328 (85.4%)		396 (84.8%)	197 (85.7%)		257 (82.9%)	131 (85.1%)	

* p value < 0.05 as statistically significant. ^a^ OR (95% C.I.): 1.637 (1.102-2.432); ^b^ OR (95% C.I.): 1.616 (1.067-2.446) The odds ratio (OR) with their 95% confidence intervals were estimated by logistic regression models.

**Table 5:** Clinical statuses and genotypic frequencies of *NUCB2/nesfatin-1* rs757081 in 1161 oral cancer patients.

				NUCB2/nesfatin-1 rs757081					
	All (N=1161)			Age < 60 (N=697)			Age ≥ 60 (N=464)		
Variable	CC (N=479)	CG + GG (N=682)	p value	CC (N=269)	CG + GG (N=428)	p value	CC (N=210)	CG + GG (N=254)	p value
**Clinical Stage**									
Stage I+II	218 (45.5%)	283 (41.5%)	0.174	111 (41.3%)	181 (42.3%)	0.789	107 (51.0%)	102 (40.2%)	0.020^*,a^
Stage III+IV	261 (54.5%)	399 (58.5%)		158 (58.7%)	247 (57.7%)		103 (49.0%)	152 (59.8%)	
**Tumor size**									
≦ T2	245 (51.1%)	324 (47.5%)	0.222	132 (49.1%)	203 (47.4%)	0.673	113 (53.8%)	121 (47.6%)	0.186
> T2	234 (48.9%)	358 (52.5%)		137 (50.9%)	225 (52.6%)		97 (46.2%)	133 (52.4%)	
**Lymph node metastasis**									
No	321 (67.0%)	449 (65.8%)	0.676	166 (61.7%)	277 (64.7%)	0.422	155 (73.8%)	172 (67.7%)	0.152
Yes	158 (33.0%)	233 (34.2%)		103 (38.3%)	151 (35.3%)		55 (26.2%)	82 (32.3%)	
**Cell differentiation**									
Well	73 (15.2%)	107 (15.7%)	0.835	40 (14.9%)	64 (15.0%)	0.976	33 (15.7%)	43 (16.9%)	0.725
Moderate or poor	406 (84.8%)	575 (84.3%)		229 (85.1%)	364 (85.0%)		177 (84.3%)	211 (83.1%)	

* p value < 0.05 as statistically significant. ^a^ OR (95% C.I.): 1.548 (1.070-2.239) The odds ratio (OR) with their 95% confidence intervals were estimated by logistic regression models.

**Table 6:** Clinical statuses and genotypic frequencies of *NUCB2/nesfatin-1* rs10766383 in 1161 oral cancer patients.

				NUCB2/nesfatin-1 rs10766383					
	All (N=1161)			Age < 60 (N=697)			Age ≥ 60 (N=464)		
Variable	TT (N=286)	TG + GG (N=875)	p value	TT (N=157)	TG + GG (N=540)	p value	TT (N=129)	TG + GG (N=335)	p value
**Clinical Stage**									
Stage I+II	129 (45.1%)	372 (42.5%)	0.443	58 (36.9%)	234 (43.3%)	0.153	71 (55.0%)	138 (41.2%)	0.007^*,a^
Stage III+IV	157 (54.9%)	503 (57.5%)		99 (63.1%)	306 (56.7%)		58 (45.0%)	197 (58.8%)	
**Tumor size**									
≦ T2	146 (51.0%)	423 (48.3%)	0.427	72 (45.9%)	263 (48.7%)	0.530	74 (57.4%)	160 (47.8%)	0.064
> T2	140 (49.0%)	452 (51.7%)		85 (54.1%)	277 (51.3%)		55 (42.6%)	175 (52.2%)	
**Lymph node metastasis**									
No	194 (67.8%)	576 (65.8%)	0.534	91 (58.0%)	352 (65.2%)	0.098	103 (79.8%)	224 (66.9%)	0.006^*,b^
Yes	92 (32.2%)	299 (34.2%)		66 (42.0%)	188 (34.8%)		26 (20.2%)	111 (33.1%)	
**Cell differentiation**									
Well	44 (15.4%)	136 (15.5%)	0.949	21 (13.4%)	83 (15.4%)	0.537	23 (17.8%)	53 (15.8%)	0.600
Moderate or poor	242 (84.6%)	739 (84.5%)		136 (86.6%)	457 (84.6%)		106 (82.2%)	282 (84.2%)	

* p value < 0.05 as statistically significant. ^a^ OR (95% C.I.): 1.748 (1.160-2.632);^ b^ OR (95% C.I.): 1.963 (1.207-3.194) The odds ratio (OR) with their 95% confidence intervals were estimated by logistic regression models.
